# Preparation, Properties and Chemical Modification Methods of the Fire-Fighting Foam for Coal Spontaneous Combustion

**DOI:** 10.3390/ma18214888

**Published:** 2025-10-25

**Authors:** Chenchen Feng, Ying Li, Hua Li, Mengmeng Bai, Zefeng Jing

**Affiliations:** 1 School of Intelligent Construction and Environment, Xi’an Jiaotong University City College, Xi’an 710018, China; 2 Key Laboratory of Thermo-Fluid Science and Engineering of MOE, School of Energy and Power Engineering, Xi’an Jiaotong University, Xi’an 710049, China

**Keywords:** fire-fighting foam, coal spontaneous combustion, chemical modification, gel foam, three-phase foam, curing foam

## Abstract

Coal spontaneous combustion causes both human casualties and environmental pollution. Owing to special flow behaviors, foam materials used in fire-fighting technology can effectively bring water and solid non-combustible substances into the fire-fighting area, greatly preventing spontaneous combustion. This paper systematically elucidates three foam materials, three-phase foam, gel foam and curing foam, and analyzes their physical and chemical inhibition mechanisms on coal spontaneous combustion. In particular, the preparation, performance and latest chemical modification methods of the foam materials are summarized in detail. It is found that foam materials with environmental friendliness, economy and excellent anti-fire performance need to be consistently explored. The primary application areas for cement-based foamed materials remain the building materials and civil engineering industries, and their modification should be studied accordingly based on the specific application context. Furthermore, a new component of foam materials, coal gasification slag (a solid waste), is proposed. In addition, the seepage properties of fire-fighting foam in porous media should be fully studied to accurately grasp the dispersion of foam materials in mine goafs. This review provides new insights and guidance for the development of fire-fighting foam materials.

## 1. Introduction

Mine disasters are the main factors affecting the healthy development of the coal industry, and coal spontaneous combustion is one of the primary disasters in the process of coal mining [1,2,3]. Besides burning a large amount of coal resources, coal fire can also induce serious disasters like harmful gas leaks and dust explosions, resulting in significant human and economic losses [4,5,6]. Underground goafs are semi-closed spaces that are filled with broken coal and rock mass. The broken coal readily reacts to oxygen in air channels, which gradually accumulates heat and eventually leads to spontaneous combustion [7]. During the mining process of shallow coal seam, the overlying rock above the goaf experiences subsidence and disturbance, resulting in the interconnection of multiple goafs to form a large composite goaf. However, preventing spontaneous combustion of coal in a large goaf through traditional methods such as grouting [8,9], inert gas injection [10], physicochemical inhibitors [11], and gel materials [12] is extremely difficult. In contrast, new anti-fire technology with foam as a carrier can form a flow in coal bodies. Compared with traditional fire-fighting technology, foam materials can play a synergistic role, which can effectively bring more water and solid non-combustible substances into the anti-fire area, greatly improving the anti-fire effect.

Foam is a heterogeneous dispersed system containing “gas and liquid (solid)” [13]. Fire-fighting foams for coal mines include two-phase and three-phase foams, inhibited foam, gel foam, organic and inorganic curing foams, etc. [14]. Conventional two-phase foams mainly consist of inert gas (N_2_, CO_2_, etc.) and surfactant solution. The main component of the liquid film is water. During the flow and diffusion of the foam, water is carried to the high-temperature fire source. The water vaporizes rapidly at high temperatures, and the vaporization will absorb significant heat from the fire zone, thereby lowering the temperature of the coal body and the surrounding environment. After foam dehydration and crushing, inert gas dilutes oxygen concentration and prevents coal spontaneous combustion [15]. However, in practical applications, two-phase foams exhibit poor stability, and thus, they are not generally used as a long-term filler.

To control coal spontaneous combustion disasters, different countries have developed a variety of materials to prevent coal fire disasters through heat absorption, cooling and oxygen isolation. Three-phase foam is based on two-phase foam with the addition of solid non-combustible materials, and then uses the foaming method to uniformly disperse the solid particles across the foam film surface, forming a triple-phase fire-fighting material. Studies [16] indicate that the solid particles added to the three-phase foam can significantly enhance foam stability. Gel foams are dispersion systems in which the colloid is formed by a crosslinking reaction between a crosslinking agent and polymer in the process of foam formation, with the colloid finally adsorbed on the foam liquid film. They have both the good diffusion properties of foam and good water retention properties of gel [17]. In addition to the above materials, the curing foam material applied in plugging technology can efficiently plug air channels, reducing the contact between coal seams and oxygen. This is also a critical technology for preventing spontaneous combustion of coal in goaf areas [18].

Unlike traditional two-phase foams, the addition of solid particles to a three-phase foam system, or adding thickeners and crosslinkers to a gel foam system, can significantly influence the foam’s interfacial properties, thus affecting its structure and performance [17]. In addition, several factors like the type of surfactant [19], the type of gels and crosslinkers used in the gel foam [20], and the proportion of components required in the formation of the foam material [21] have influences on the performance of the resulting fire-fighting foam material. When selecting foam materials for fire-fighting projects, it is also necessary to consider their economy. For example, in the preparation of three-phase foams, cheap fly ash particles are usually used instead of nanoparticles (silicon dioxide, aluminum oxide, aluminum hydroxide, etc.) to reduce their cost, while for gel foams, gel control is relatively complex due to the relatively expensive preparation of colloidal materials. Therefore, many scholars have continuously explored the various components of gel foam and their best combinations, and used various physical and chemical methods to improve its fire resistance. In recent years, with the increasing concern about environmental problems, some new environmentally friendly gel foams have also received people’s attention [22].

In addition to the above contents, the diffusion behaviors of fire-fighting foam within coal and rock media of the large goaf space are also key to its application in the field of fire-fighting. As a non-Newtonian fluid, foam exhibits highly complex seepage and diffusion mechanisms within the heterogeneous pore structure of coal seams. The seepage characteristics of foam fluid are closely related to the rock pore structure and foam structure [23,24]. When foam fluid is continuously injected into a pore space, the structure of the foam changes with time. The fractured rock in the mined-out area is at low pressure and is mostly in a state of natural accumulation. The seepage mode of foam fluid in the mined-out area is obviously different from that of foam fluid in the reservoir primary rock. Hence, it is necessary to conduct systematic research on the seepage and diffusion properties of fire-fighting foam fluid in the porous media of coal seams.

Based on the above problems, this paper systematically reviews the research status of several foam materials in mine fire-fighting technology, including three-phase foam, gel foam and curing foam. The preparation methods, properties and modification methods for each foam material are summarized in detail, and meanwhile, the existing problems and development directions of these materials and their corresponding fire-fighting technologies are discussed. The paper presents ideas for the development of fire-fighting foams.

## 2. Three-Phase Foam

Three-phase foam is a foam system that consists of three phases (gas, liquid, and solid), stabilized by the adsorption of particles at the gas–liquid interface [19]. Solid particles, like silica [25,26,27], CaCO_3_ [28] and alumina [29], are commonly used to stabilize foam systems. However, due to high cost [30] and agglomeration problems [31], the application of foam stabilized by nanoparticles is limited. Three-phase foams in fire-fighting in coal mines mostly use fly ash and yellow mud (the main component is clay minerals) as the solid base materials [30]. Fly ash and yellow mud have wide sources and low prices, making them suitable for preparing three-phase foam. Meanwhile, coal gasification slag, a solid waste in the coal chemical industry, can also be effectively used as the solid component of three-phase foam. Unlike two-phase foam, the presence of solid particles on the liquid film allows the formation of a relatively stable skeleton structure within the three-phase foam. This structure maintains foam stability for a long time and effectively hinders the adsorption of oxygen on the coal surface. After the foam bursts, the non-combustible particles in the foam remain attached to the surface of the abandoned coal, effectively covering the oxygen and preventing spontaneous combustion [32].

**Figure 1:**
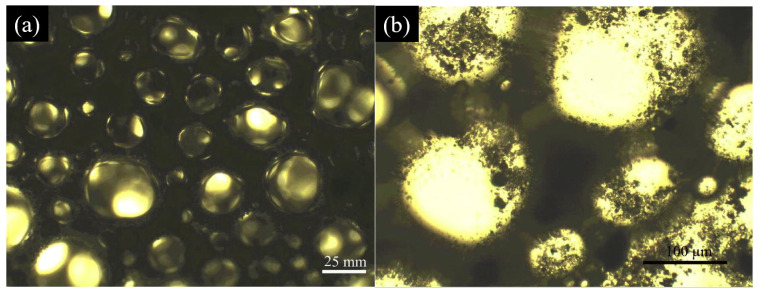
(**a**,**b**) Optical micrographs of a three-phase foam stabilized by fly ash particles of 33 wt.% [32].

In 1995, Michaylov at Sofia University prepared a three-phase foam composed of fly ash, nitrogen and water for the first time, and conducted a study on the prevention of mine fires in the Bobov Dol coal field, which achieved good results [33]. Subsequently, the three-phase foam was successfully deployed in fire-fighting operations such as the Baijiaogou Coal Mine fire in Ningxia, China [30]. Wang et al. [32,34] successfully prepared fire-fighting three-phase foam. They also combined fly ash, nitrogen and water through physical and mechanical foaming (Figure 1), and studied the optimal concentration ratio between the slurry and foaming agent. Subsequently, a few scholars [35,36,37] also carried out studies on the formation mechanisms, microstructures, inhibition mechanisms, material properties and preparation methods of three-phase foams, and accumulated a lot of theoretical achievements and practical application experience.

### 2.1. Formation Mechanism and Microstructure

**Figure 2:**
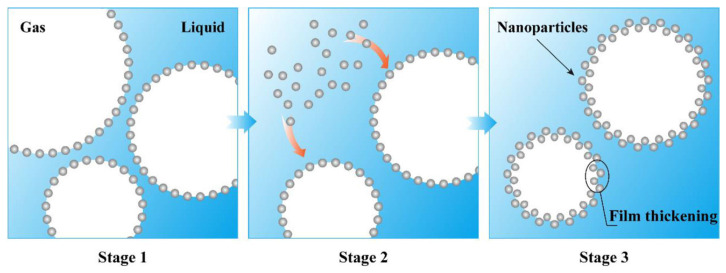
Diagram of the mechanism of microparticle-stabilized foam [50].

Studies suggest that the stabilizing mechanism of foam containing particles includes two aspects: interactions between the liquid film and particles and interactions between particles [38]. One important mechanism is the adsorption of particles on the liquid film, as shown in Figure 2. Detachment energy is required for particle desorption from the gas–liquid film. As the detachment energy increases, the adsorption of particles on the film increases, the drainage rate of the liquid film decreases, and thus the foam stability increases. Relevant studies [25] indicate that at the optimal nanoparticle mass fraction (0.06% silica), adding silica nanoparticles to aqueous solutions can extend the foam half-life from 80 min (sodium dodecyl sulfate solution) to 120 min. The average bubble size of foam containing 3 wt.% silica nanoparticles is 625,466 μm^2^, approximately 6.3 times larger than that of foam containing 0.5 wt.% silica nanoparticles (3,950,377 μm^2^). As the concentration of silica nanoparticles increases, the number of bubbles rises while their size gradually decreases. The detachment energy is associated with particle diameter, gas–water interfacial tension, and contact angle between particles and the gas–liquid interface [35]. The adhesion of solid particles on the interface enhances the strength and elasticity of the liquid film, reducing gas diffusion between bubbles [39,40,41]. In addition, the highest pressure that the liquid film between bubbles can bear (maximum capillary pressure) is crucial in preventing foam accumulation [36]. If the bubbles have a coalescence tendency, it is necessary to overcome the capillary pressure formed by the adsorption of microparticles. Compared with the two-phase foam, the presence of solid particles on the liquid film increases the maximum capillary pressure of the bubbles, resulting in greater foam stability. [37,42,43]. The maximum capillary pressure of bubbles stabilized by solid particles is related to particle radius, gas–liquid interfacial tension and the contact angle between the particles at the gas–liquid interface [44,45].

**Figure 3:**
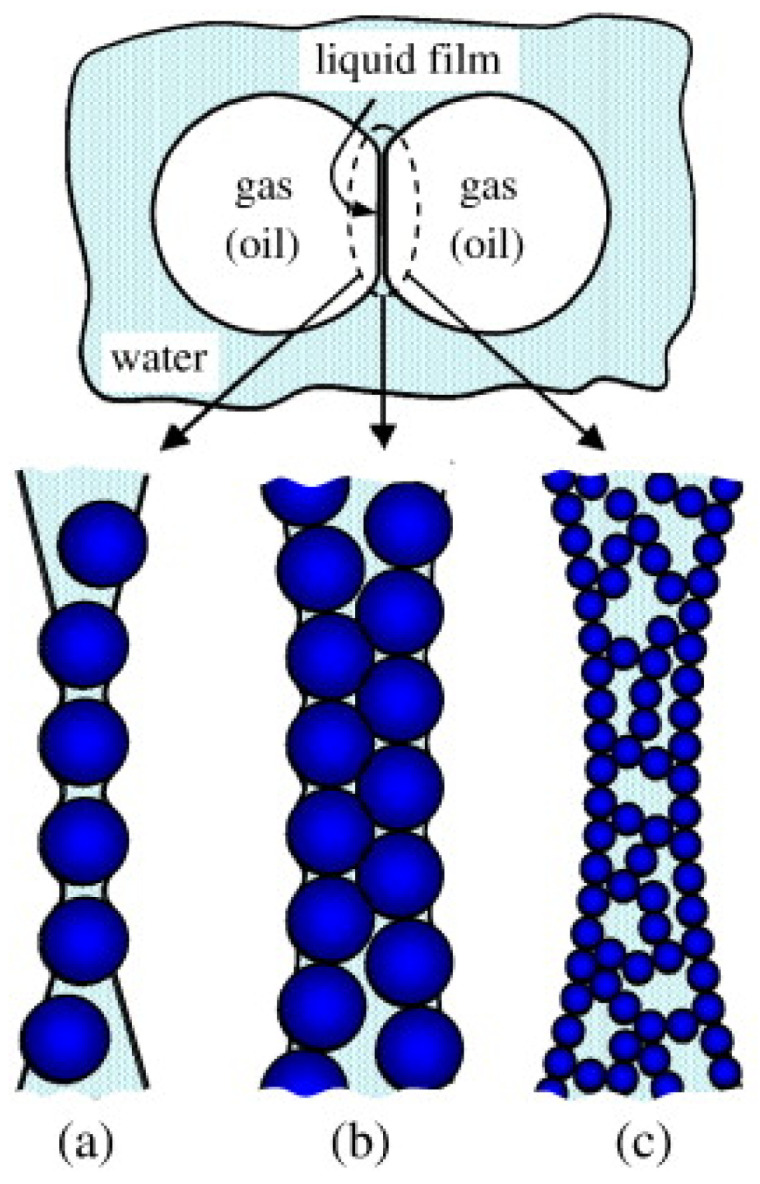
Mechanisms of solid particle-stabilized liquid films: (**a**) single-layer bridged particles; (**b**) double-layer enclosed stacked particles; (**c**) particle aggregate network [38].

The interaction between particles is another crucial mechanism for foam stability, which can create an overall network structure and allow bubbles to separate. These interactions reduce the drainage of liquid films, thereby minimizing bubble coalescence and prolonging the existence time of the system [46,47]. Figure 3 illustrates the network structure formed by nanoparticle interactions. The nanoparticles dispersed in the foam solution can form three kinds of structures: single-layer structures, thick multilayer structures and network structures [38,48]. Due to their high detachment energy, the nanoparticles will accumulate on the liquid film, slowing down the drainage rate. As the liquid film thins, the particles rearrange, resulting in the transformation of a double-layer into a single-layer structure. A tightly packed monolayer of particles on the surface of the film resists resistance, thereby slowing film thinning and preventing the film from breaking through the bridging monolayer [49]. For these three potential structures, a general formula for the maximum capillary pressure was developed, encompassing these particle arrangements [36]:

(1) $$p_{c}^{\max}=p\frac{2\sigma }{R}(\cos\theta +z)$$

where *p*_c_^max^ is the maximum capillary pressure; *p* = 2*f*, *f* represents the projected area ratio of the particles to the total interface area. The *f* for the hexagonal close-packing structure is 0.907; *σ* is the liquid–gas interfacial energy; *R* is the radius of the spherical particle; *θ* is the contact angle between the liquid and solid particle in a gas environment; *z* is a constant depending on the particle arrangement within the liquid film. *z* is 0 for a monolayer of particles. *z* is 0.633 for a closely packed bilayer of particles. Research on the stabilizing effect of solid particle networks on emulsion and foam indicates that the contact angles between solid particles and liquid films are different for foams stabilized by a single layer and a double layer of particles [36].

### 2.2. Influencing Factors for the Properties of Three-Phase Foam

The three-phase foam in the oil industry uses nanoparticles as the solid phase and CO_2_ or N_2_ as the gas phase [51] to improve oil recovery [52,53]. For the three-phase foam used for coal fire-fighting and control, fly ash and yellow mud are generally used as the solid base material, and N_2_ is used as the gas phase [32], which aims to ensure safe coal mining operations. Some scholars compared N_2_-CO_2_ foams stabilized by silica nanoparticles with CO_2_ foams, analyzed their growth mechanisms, and concluded that the coarsening rate of N_2_-CO_2_ mixed foams is similar to that of CO_2_ foams. Regardless of the gas composition, the nanoparticles determine the foam properties, meaning that nanoparticles determine the interface behavior on the gas–liquid interface [54]. Therefore, we can assume that the parameters of the solid particles have more influence on the properties of the three-phase foam than the gas type.

**Figure 4:**
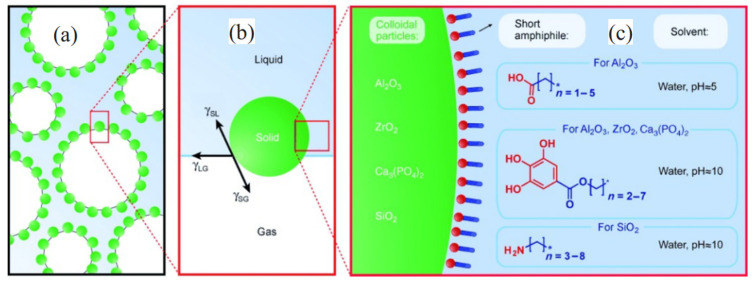
Potential methods to attach colloidal (solid) particles at gas–liquid interfaces by tuning their surface-wetting properties. (**a**) Schematic diagram of colloidal (solid) particles stabilizing gas bubbles. (**b**) Adsorption of hydrophobic particles at the gas–liquid interface. (**c**) Methods for adjusting the wetting properties of originally hydrophilic particles [55].

The adhesion of particles on the gas–liquid interface requires an optimal balance among solid–liquid, solid–gas and liquid–gas interfacial tensions [55,56], as shown in Figure 4. Therefore, the hydrophobicity of solid particles is a key factor affecting the liquid film stability. The hydrophobicity of solid particles affects the contact angle between solid particles and the liquid phase [57]. Studies [55] have shown that when the contact angle of nanoparticles is approximately 90◦, their detachment energy can reach more than 1000 *k*T, which is higher than that of surfactants (where *k* is the Boltzmann constant, whose value is 1.3806505 × 10^−23^ J/K, and T represents the Kelvin temperature). High detachment energy can make the adsorption of nanoparticles on the gas–liquid film irreversible [35]. Generally, solid particles possessing varying degrees of wettability demonstrate distinct stabilization effects on foam. In addition, the synergistic effect between nanoparticles and surfactants is also important for foam stability. When hydrophobic or hydrophilic nanoparticles are mixed with surfactants at the optimal concentration, they can play a positive role in stabilizing the foam [28,43,58]. Many methods have been reported in the literature to modify the wettability of solid particles to facilitate their adhesion on the gas–liquid interface, thus forming stable foams [59].

Nanoparticles used in three-phase foam have the characteristics of regular particle shapes and a single composition. Nevertheless, the three-phase foam applied in fire-fighting for coal mines mostly uses fly ash and yellow mud as solid base materials. Nowadays, in China, a large number of coal gasification slags can also be an alternative. The shapes of these solid particles are irregular and the compositions are complex. Fly ash-stabilized three-phase foam differs from that stabilized by nanoparticles. More complex interface behaviors will occur between non-uniform particles and between solid particles and the gas–liquid interface. Some studies on fly ash-stabilized three-phase foam show that the concentration, size, and hydrophobicity of solid particles have a great influence on the foam stability [60]. As particle concentration increases, the foam stability first increases and then decreases. As particle size decreases and hydrophobicity increases, the stability of the three-phase foam increases [38,48,60]. However, studies related to three-phase foam containing coal gasification slag are lacking. Thus, this could be a development direction in the future.

In addition to the stability of the three-phase foam itself, attention must also be paid to its seepage and diffusion ability within the porous medium of the coal seam. The seepage characteristics of the foam fluid are closely related to the rock pore and foam structure [61,62]. When the foam fluid is continuously injected into pores, the structure of the foam changes with time [63]. When bubbles migrate into pores of comparable size, the surface tension of the bubble interface plays a dominant role, and the gravity-induced drainage of the liquid film and the bubble coalescence caused by the thinning of the liquid film will be significantly reduced. In this case, the foam in the pore space will gradually lose its original structure due to the diffusion of gas between bubbles; that is to say, the structural evolution of bubbles is mainly accomplished through the coarsening process [64,65]. At present, research on the mechanism of diffusion of foam in porous media is mainly aimed at the process of foam coarsening [66,67,68]. Jones et al. [69] investigated the coarsening process of bubbles in a two-dimensional micromodel of irregularly hexagonal etched borosilicate glass, and they found that the bubbles injected into the model rapidly coarsened and reached the pore size. Ultimately, the liquid film stabilized within an area of flat curvature.

**Figure 5:**
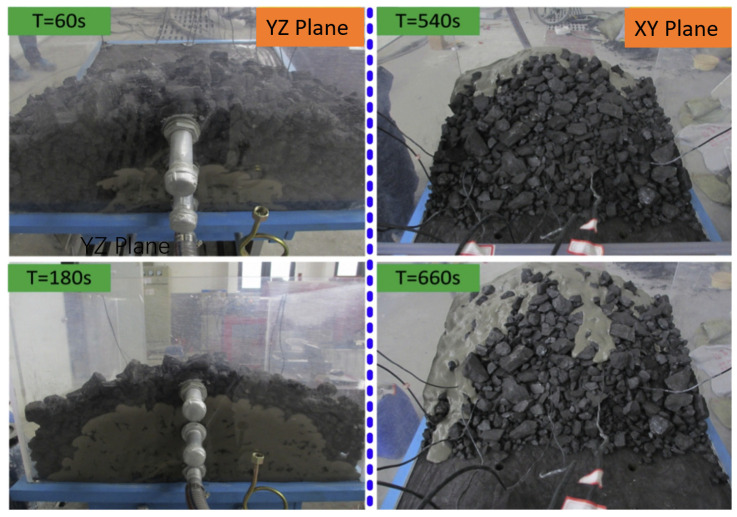
Flow and sealing effect of aqueous foams stabilized by microparticles in fractures [16].

At present, the research on three-phase foam fluid flow in the porous medium of coal and rock is mainly based on macroscopic experiments. Lu et al. [16] established a similar simulation platform to investigate the diffusion effect of particle-stabilized foam fluid in fracture channels with varying inclinations and azimuth, and obtained the fitted experimental parameters. Different from ordinary cement slurry, the diffusion of foam fluid in the Y- and Z-directions appears at the same time, enabling thorough accumulation and permeation throughout the entire loose gangue pile, as shown in Figure 5. Qin et al. [32] used carboxymethyl cellulose (foam stabilizer), nitrogen, fly ash particles and sodium dodecyl benzene sulfonate to prepare three-phase fire-fighting foam, and proved via experiments that the three-phase foam had good fire-fighting performance. The foam prepared by them is especially suitable for preventing the spontaneous combustion of large areas of pulverized coal or extinguishing fires at a higher location in the goaf area. Wang et al. [19] optimized the fly ash-stabilized three-phase foam system. The optimized foam system was composed of an anionic surfactant *α*-olefin sulfonate with a mass fraction of 0.5%, suspension agent with a mass fraction of 4%, and fly ash with a mass fraction of 8%. They conducted experimental studies on the ability to seal the reservoir fracture channel of the optimized three-phase foam. Results showed that the optimized foam system exhibited sealing performance over 20 times greater than normal foam. Therefore, fly ash-stabilized three-phase foam is an effective sealing system.

Unlike porous media of low permeability, rock fractures in mine goafs have less pressure and are mostly in a state of natural accumulation, and the three-phase foam flow pattern within the fracture channels is obviously different from that of foam fluid in an oil reservoir. Goafs are characterized by large porosity and large seepage channels. In this case, the size of the bubble will be much smaller than the pore size. Will the foam structure mainly evolve through coarsening in this situation? How will this change affect its percolation and accumulation characteristics in porous media? At present, research on foam seepage in porous media is mainly focused on the petroleum industry. Regarding the fire-fighting of coal mines, research has mainly evaluated the fire-fighting performance of foam. To provide new insights for predicting the seepage characteristics of three-phase foam and achieve precise directional control of foam flow in porous media, it is essential to further study the structural evolution characteristics of foam within porous media from a microscopic perspective.

## 3. Gel Foam

Gel foam is a dispersion system created by a foaming solution, crosslinking agent and polymer under the action of gas, with gas uniformly dispersed throughout the gel. The gelling agent in the foam is crosslinked to form glue under the action of a crosslinking agent (e.g., sodium bicarbonate) [70]. Firstly, the foaming agent is mixed with water through a proportional mixer, then high-pressure inert gas is passed into the foam. Finally, the thickening gelling agent is added to the foam liquid. The foam surface is continuously crosslinked under the action of crosslinking, finally forming a gel with a three-dimensional network structure [71]. Gel foam has a unique formation process that allows it to combine the benefits of both gel and foam. In contrast to water-based foam, gel foam exhibits significantly stable water retention properties. After gelling, a protective film is formed on the coal surface to cool down and cut off oxygen. Compared with traditional gels, gel foam materials are characterized by their large foam volume, high fluidity, low price and large coverage [21]. At present, researchers are using advanced physical and chemical methods to develop a variety of effective gels and composite pastes with different inhibition mechanisms. Gelling mechanism [72,73], fire extinguishing efficiency, inhibition properties of gels and slurries [73,74], inhibition mechanism [75,76] and other aspects have received more and more attention [77,78]. The above fire-extinguishing agents are significant for further exploring new technologies in preventing coal spontaneous combustion.

### 3.1. Inhibition Mechanism

According to a large number of experimental studies, the mechanisms by which gel foams prevent coal spontaneous combustion are as follows: (1) Cooling [20]. When the gel foam is delivered to the high-temperature area, the water within the foam rapidly evaporates, absorbing a lot of heat and lowering the temperature of the coal. (2) Isolation from oxide [79,80]. The gel foam contains gels and crosslinkers, and after a chemical reaction, a three-dimensional network structure is formed and covers the coal surface. When the water has completely disappeared, a gel membrane forms, preventing oxygen from contacting the coal. (3) Physical and chemical suppression [81]. Physical suppression is typically employed to keep oxygen from contacting coal. Chemical inhibition prevents the oxidation reaction of coal by replacing the free radicals involved in the oxygen reactivity of coal, or producing more stable structures to prevent the oxidation of coal. Throughout the whole process of gel slurry diffusion and permeation within loose coal, the skeleton of the loose coal will play a great role in filtering the gel particles.

### 3.2. Components of Gel Foam

The components of gel foam are mainly a gelling agent, crosslinking agent, foaming agent and gas. At present, gel foam is primarily divided into silicate gel foam, acrylamide copolymer gel foam and natural polymer gel foam.

The primary gelling agent used in silicate gel foam is water glass [73]. The mechanism of action for both water glass gel foam and water glass gel involves a chemical reaction between the gel agent and the crosslinking agent. The difference lies in the foaming agent added to the gel foam, which forms a network structure on the foam film. Water glass gel foam has a low price and resistance to high temperatures. However, it also suffers from issues such as low foam stability and strength.

Acrylamide is a common material for preparing gel foam [82]. Polyacrylamide is a widely used polymer whose molecule contains an amide group that facilitates hydrogen bonding, making it a widely used polymer. It has excellent chemical reactivity and water solubility, enabling crosslinking or grafting to produce various modified agents with branched or network structures. After modification, the fire resistance, strength and temperature resistance of acrylamide copolymer gel foam can be improved. However, due to the high requirements of the preparation process and acid–base stability, its application is limited [83,84].

Natural polymer-based gel foam uses cellulose or other natural polymers as the gelling agent [85]. The raw materials used for polymer gel foams come from a wide variety of sources. They are naturally degradable and thus less harmful to the environment. Additionally, natural polymer foams are highly susceptible to microbial degradation during storage due to their composition serving as a natural “food source” for microorganisms. This degradation leads to reduced strength and diminished stability. Such degradation severely compromises their long-term usability and emergency reliability as reserve materials in mine fire prevention and suppression, posing significant safety risks. As a result, they have been focused on by a few researchers in recent years [71,86,87]. However, while the stability of current biomass gel foam has been significantly enhanced, the impact on the expansion ratio is often overlooked [71]. Meanwhile, the foams that form after dehydration and drying exhibit high porosity and low thickness, making it difficult to realize a complete oxygen barrier [21]. Therefore, their application is limited, and modifications are expected to be the primary direction of future studies on the development of natural polymer-based gel foams.

Different colloidal materials have different inhibition properties for spontaneous combustion of coal, but most colloidal materials have good water retention, thermal stability and sealing effects. Compared with other suppressants such as ionic liquids and conventional foams, colloids have relatively low mobility, which allows colloidal materials to cover coal pore spaces. However, compared with the three-phase foam, the preparation of colloidal suppression materials is relatively expensive, the gel control is relatively complicated, and the coverage of gel foam is limited [88].

**Figure 6:**
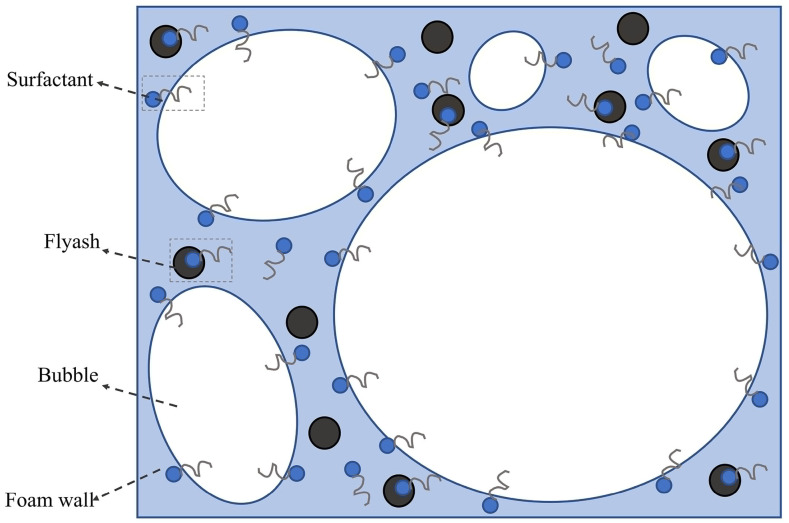
Adsorption of aggregates on suspended gel foam films [17].

As shown in Table 1, we summarized the studies of the gel foams. Many scholars have explored the best combinations of composite foaming agents, optimized the proportion of each component in gel foams, and studied their thermal stability, blocking performance and fire-fighting effects (Table 1). A few studies have shown that adding fly ash [70], corn straw [88], bentonite [89] and other suspended aggregates into hydrated slurry causes the hydrophilic side of surfactants to bind with the aggregate particles while the hydrophobic side remains exposed in the liquid phase. This improves the surface viscosity and stability of the foam (Figure 6). After the water in the gel is exhausted, the residue creates a barrier layer over the coal surface, blocking contact between oxygen and coal, and extending the inhibition period. In addition, more and more environmentally friendly and economical substrates or fillers, such as biomass [85,87], are used in the preparation of gel foams. Most of them originate from by-products or waste materials generated by agriculture, forestry, or the food processing industry, so they can mitigate risks to the environment. The fire-fighting properties of these novel gel foam materials are still of concern.

### 3.3. Diffusion Characteristics in Porous Media

**Figure 7:**
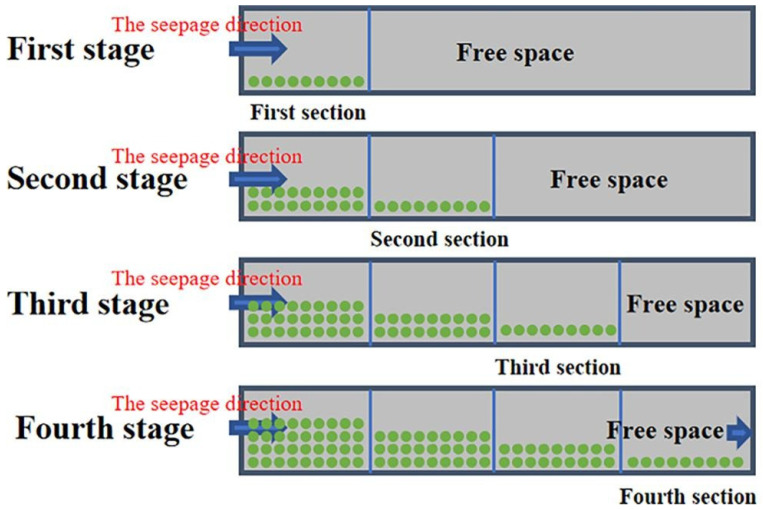
Evolution of filtering effect as the inorganic gel seeps and diffuses in loose coal particles [99].

Similar to the three-phase foam, the diffusion characteristics of gel foam in the porous media of the coal body need to be focused on. During the diffusion of gel slurry within loose coal media, the skeleton of the loose coal will play a great role in filtering the gel particles [99], as shown in Figure 7. Gel particles filter through the skeleton of coal particles and ultimately adhere to the coal surface. The precipitation and adsorption of the gel particles eventually result in a decrease in gel diffusion rate, inducing the gradual blockage of the pores. The time of grouting and the distance of diffusion are highly related to the permeability coefficient.

When gel foam flows into porous media, they are subjected to three forces [77]: grouting pressure force, gravity and resistance. Resistance refers to the frictional force between the foam and the coal–rock fissure, with the gel foam diffusing through the loose coal under the action of the resultant forces. From the flow distribution of gel foam in porous media [77], it can be seen that the porosity ratio of coal rock in the mining area gradually increases from top to bottom, with lower resistance in the lower section than in the upper section. The gel foam has a faster horizontal diffusion speed, with an elliptical diffusion peak. Gel foam flows toward the minimum pressure direction within porous media. During the initial grouting phase, the gel has not formed, resulting in low viscous force and good fluidity. As the gel content increases, the viscosity rises, and the fluidity of the gel foam becomes worse, causing the gel foam to accumulate mainly vertically. Notably, when the gel foam flows toward the boundary, the backflow takes place and accumulates in a vertical direction until it fills the entire space.

**Figure 8:**
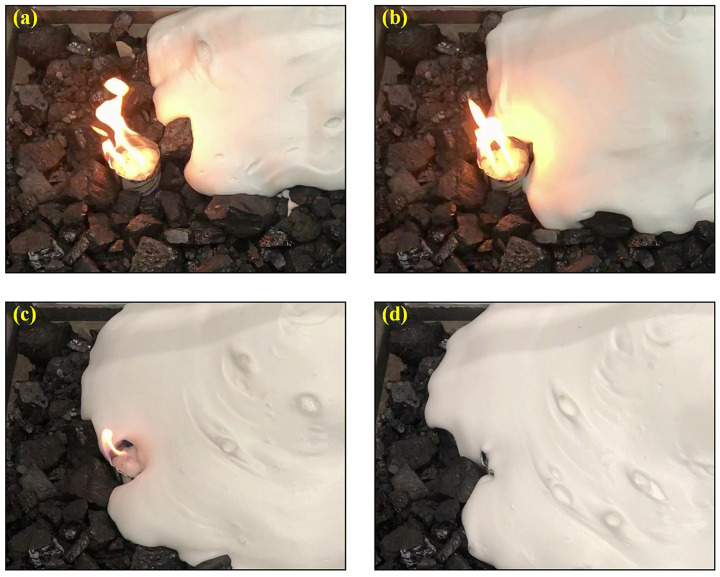
(**a**–**d**) Coverage and extinguishment process of gel-stabilized foam fluids to the open coal fire [15].

Shi et al. [15] investigated the flow properties of gel foam in loose coal by using a simplified coal-filling experimental platform. Their study has shown that the gel foam has a hemispherical dispersion form within porous medium, enabling the foam fluid to quickly cover and extinguish open flames while demonstrating superior thermal stability, as shown in Figure 8. In addition, the foam fluid effectively captures the concealed fire in the porous mediums through rapid permeation, ultimately cooling the hot coal fire after an injection of the foam, inducing the decrease in temperature from 760 °C to 30 °C.

Fire-fighting technology of gel foam can effectively keep coal from spontaneous combustion, thus maximizing the economic benefit of coal mines [73,100,101,102]. However, the gel foam materials developed at present still suffer from drawbacks of low foam expansion rate, long gel formation time, and low stability [17]. Therefore, it is of great significance to investigate novel foam gel to further enhance the fire-retardant and extinguishing performance [12,103,104]. At present, the fire-fighting technology of gel foam has successfully prevented many coal spontaneous combustion disasters in China. However, the following problems still need to be solved in gel foam fire-fighting technology [99]: (1) the micro-formation mechanism for gel foam; (2) the evolution of gel foam structures and their correlation with foam stability; (3) mechanical model analysis of gel foam’s structural stability; (4) flow pattern of gel foam in porous media.

## 4. Curing Foam

**Figure 9:**
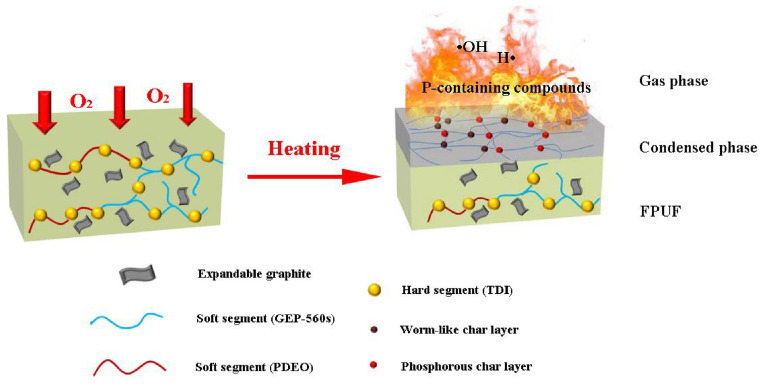
Schematic for flame-retardant mechanism of FPUFs (flexible polyurethane foams) [106].

Gel foams exhibit poor stability and low strength, with water loss easily causing the foam film to burst [71,73,84,105]. Research shows that plugging and sealing technology can efficiently block air leakage channels, minimize oxygen contact with coal seams, and serve as a critical technology for preventing spontaneous combustion in goaf areas [106], as shown in Figure 9. Organic curing and inorganic curing foams are often used to fill abandoned roadways. Organic curing foam features a short gelling time, good stability and high stacking ability, but it also has many drawbacks, such as high exothermicity, toxicity, easily causing coal spontaneous combustion, and high cost, which seriously limit its application in mines [107]. In contrast, inorganic curing foams are non-combustible, generate less heat, and are low-cost, but they commonly suffer from drawbacks of prolonged gelling time, low stability, and poor stacking capacity, so the abandoned roadway cannot be filled quickly and stably.

### 4.1. Inhibition Mechanism

Organic curing foam is a leakage-plugging material which is a mix of resin, foaming agents and curing agents according to specific proportions [108]. Common organic curing foams include polyurethane foam, phenolic foam, urea–formaldehyde foam, and cured polymer composite foam. They have excellent sealing properties [109]. A comparison of the properties of organic curing foams is shown in Table 2. Toughening of foams and catalytic curing of resins represent the primary research directions for organic curing foams in coal mine applications.

The resin-catalyzed curing system is mainly composed of the curing agent, foaming agent and surfactant. The foaming system of the resin has a great influence on the expansion ratio, foaming ratio, microstructure and mechanical performance of the foam. A curing agent acts as a catalyst that promotes the curing reaction. After the introduction of the curing agent into the resin system, various dehydration condensation reactions will occur between the hydroxymethyl phenol in the resin molecular structure and the active hydrogen on the phenol hydroxyl group. At the same time, the resin is gradually thickened under the effect of emulsification, forming a crosslinked and interpenetrating network structure, and expanding under the action of the foaming agent to form an organic curing foam [122]. For the low-temperature foaming required for mine applications, the curing agents are mainly inorganic and organic acids. Inorganic acid curing agents are characterized by fast reaction speed, short curing time, but corrosive behavior, while organic acid is less corrosive, but the foaming is unstable and easy to contract [123].

The surfactant added to the foamed resin primarily increases the miscibility of raw materials in the resin, and the surfactant can greatly reduce surface tension, resulting in a more homogeneous and stable foaming process. There are a few types of surfactants, including non-ionic, ionic and amphoteric surfactants. The surfactants used in different types of foam vary greatly, and non-ionic surfactants are often used in phenolic foam [124,125,126]. Silicone surfactants are widely used as a foam stabilizer for polyurethane foam [127], while modified alkyl glycosides (APG) and sodium dodecyl sulfonate (SAS) are used to improve the surface tension of urea–formaldehyde foam to increase the resin compatibility [128].

The role of the foaming agent is mainly to cause the resin to gradually expand during the curing process, resulting in the formation of foam [106]. At present, foaming agents are classified into physical and chemical foaming agents. The physical foaming agent is mainly responsible for the transformation of the substance between solid, liquid and gas. It expands and forms more pores during the curing process of the resin. The chemical foaming agent undergoes chemical reactions during the curing reaction. It produces bubbles and thus the resin forms a cavity structure [129]. The preparation of organic curing foams usually uses physical foaming agents, such as petroleum ether, hydrofluorocarbons, chlorofluorocarbons, dichloromethane and so on [130,131,132]. These substances are used as foaming agents because they are liquid at room temperature and can be better dissolved into the resin system. Foam made from them has the advantages of excellent plasticity, complete cell structure and good expansibility.

Available polymer foams for mining, such as phenolic foam, phenolic aldehyde foam and urea–formaldehyde foam, suffer from issues including poor strength and toughness, and the confined plugging is not sustainable [114]. This limits the application of mining polymer foam. At present, there are two main ways to modify and toughen polymer foams for the mining: one is to add a variety of polyalcohol toughening agents and different kinds of rubber products through physical methods, and the other is to add cashew nut shell oil [133], lignin [134], linseed oil [135,136], nanosilica or titanium nitride nanoparticles [137,138] into the resin system. The primary principle of this toughening agent is that its internal flexible group and the hydroxyl group on the branch chain of the macromolecular resin are dehydrated, condensed, crosslinked and cured to achieve chemical toughening [119,129,131,139,140,141]. These modifications generally enhance flame retardancy and compressive and flexural strength. The type of chemical modifier, along with the type, size and concentration of additives, determines the extent of performance enhancement [142]. In fiber-reinforced approaches, the primary influencing factors are the compatibility between fibers and the resin matrix, along with rigidity and concentration. The flame retardancy, thermal stability, and compressive strength of nanoparticle-toughened foam materials depend on the shape, size, and surface characteristics of the nanoparticles [143].

Organic curing foams are particularly suitable for situations where rapid filling and plugging are required, the underground is impassable, and the material or equipment cannot be transported [144]. Halogen-free polyurethane materials can effectively enhance coal’s load-bearing capacity and reduce smoke emissions [145]. However, organic curing foam also has some limitations. For example, it has pyrolytic behavior during production [146].

### 4.2. Inorganic Curing Foam

Inorganic curing foam (ICF) is a plastic material formed by mixing water-based foam with composite slurry. The initial fresh state is a foam fluid, and after condensation and solidification, it becomes a porous foam. Its formation process includes the following steps: (1) the formation of water-based foam is firstly conducted; (2) during the mixing process, particles in the composite slurry adhere to and cover the liquid film of the water-based foam; (3) the particles in the liquid film are modified and the foam is stabilized; (4) the foam fluid changes from the fluid state to the curing state. Compared with the organic curing foam, inorganic curing foam has strong compression resistance and can block the air leakage channel for a long time. Inorganic solidified foam is a type of cement-based foam material, with foamed cement and foamed concrete being common examples. Cement-based foam material is often applied in the floor heating insulation layer, the roof, or the wall.

**Figure 10:**
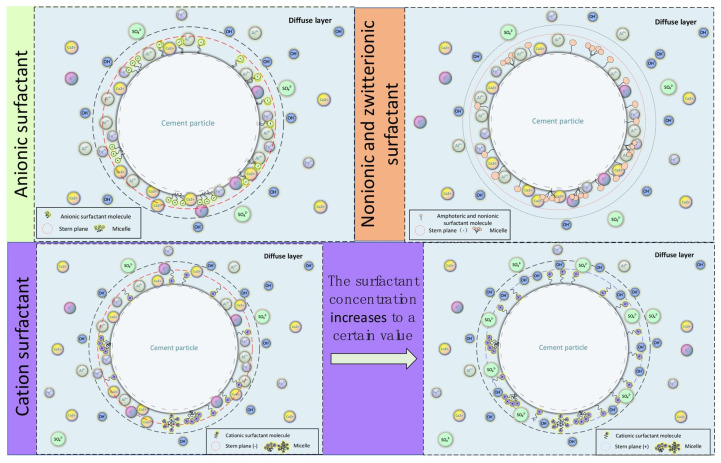
Adsorption patterns of different surfactant molecules on cement particles [150].

The performance of foaming agents determines the quality of cement-based foam materials. An excellent foaming agent is a key factor in preparing high-quality cement-based foam [147]. Figure 10 shows the pattern of cement particle adsorption onto different surfactant molecules. Research by Feneuil et al. [148] revealed that surfactants can change the yield stress of fresh cement slurry, with a reduction in yield stress being beneficial to the foam concrete’s stability. Research by Hou et al. [149] revealed significant differences in the adsorption capacity of cement particles toward various surfactant molecules. Furthermore, when foams are prepared using different surfactants, the resulting foams also display substantial variations in their pore structure. Xu et al. [150] used the Zeta potential method to study the law of cement particle adsorption for various surfactants, and tested the compressive strength, expansion rate, accumulation capacity and coal spontaneous combustion resistance of inorganic curing foams prepared by various surfactants. They found that anionic surfactant molecules exhibited the highest adsorption capacity on cement particles compared to other surfactant molecules, and it was found that the ICF prepared from surfactants with large adsorption capacity had high compressive strength, low expansion rate and poor accumulation characteristics.

As a new filling and plugging material for goafs, inorganic curing foam features oxygen insulation, cooling and high compressive strength, but it also suffers from poor stability and weak stacking ability. Several researchers have attempted to enhance the ICF’s stability through the addition of materials such as fibers and volcanic ash [151,152]. Mineral admixtures [153] usually have a great influence on the properties of foamed cementing materials. Blast furnace slag, fly ash, and silica fume are several types of widely employed industrial wastes possessing pozzolanic activity [154,155,156,157]. These materials contain substantial amounts of active SiO_2_ and Al_2_O_3_. These oxides primarily exist in an amorphous (glass-like) form. They are unstable and highly energetic, making them chemically active and readily reactive. Nowadays, coal gasification slag can also provide an alternative. The recycling of these industrial wastes helps to reduce the consumption of raw materials and the emissions of greenhouse gas. They all have a high content of silica and amorphous alumina, and they react with Ca(OH)_2_ in water. The hydration process of cement is influenced by the pozzolanic effect, and thus changes the material’s pore structure [153]. The effects of different mineral admixtures on the performance of foamed concrete are shown in Table 3.

Increasing the stability of inorganic curing foam through the addition of materials, like volcanic ash and fiber, is very limited and fails to fundamentally resolve issues like Ostwald maturation [158], coalescence [159,160,161], and gravity-driven drainage [162,163]. Research indicates that the chemical and physical properties of prefabricated foams govern the size, distribution and interconnectivity of bubbles in inorganic curing foams [164]. At present, some scholars use prefabricated foam to enhance the stability of inorganic curing foam, which may provide new ways to develop fire extinguishing materials.

**Table 3:** Effects of different mineral admixtures on the performance of foamed concrete.

Ref.	Admixture	Properties
[165]	Expanded vermiculite powder and silica powder	Silica powder enhances the compressive strength of materials. Foam concretes incorporating expanded vermiculite powder exhibit promising prospects in thermal conductivity.
[166]	Rice husk	Rice husk ash exhibits higher pozzolanic activity than fly ash, enhancing the degree of hydration. Under various preset pressures and airflow velocity conditions, the average blocking efficiency of the novel inorganic curing foam was 8.1–18.1% higher than that of traditional inorganic curing foam.
[153]	Fly ash (FA), blast furnace slag (BFS) and silica fume (SF)	When the blast furnace slag content is 20%, the foam content is twice that of the cementitious slurry, and the water–cement ratio is 0.5, the overall performance of cement-based foam material (CBFM) can be optimized. CBFM with BFS as a mineral admixture has a more uniform closed-cell structure than FA and SF.
[120]	Blast furnace slag	The activator dissolves the glassy structure of slag, generating more hydration products. This enhances the compressive strength of cement-based foam materials and further improves structural density.
[167]	Fly ash, granulated blast furnace slag (GBS)	The foam concrete with 100% GBS at a water-to-binder ratio of 0.68 exhibits better performance than the reference foam concrete.

During the foaming process, the porosity of foamed concrete is directly related to the incorporation of gas and cement-based suspension medium during the foaming process. However, the stability of the wet foam before the solidification determines the size of the pores. Wet foam undergoes a continuous Ostwald maturation and coalescence process, thereby reducing the total amount of free energy in the system [160]. These instability procedures dramatically increase bubble size, leading to big pores in the ultimate structure. Therefore, stabilizing the bubbles contained in the liquid or the initial suspension is crucial. According to earlier literature [38,55,168], colloids and other particles can adsorb onto the bubble surface, reducing the interface energy, thereby improving foam stability. Besides increasing the concentration of particles in the initial suspension and reducing the size of particles, reducing the time required for particles to diffuse and adsorb on the surface of the bubble is also conducive to the formation and stability of the foam [161].

**Figure 11:**
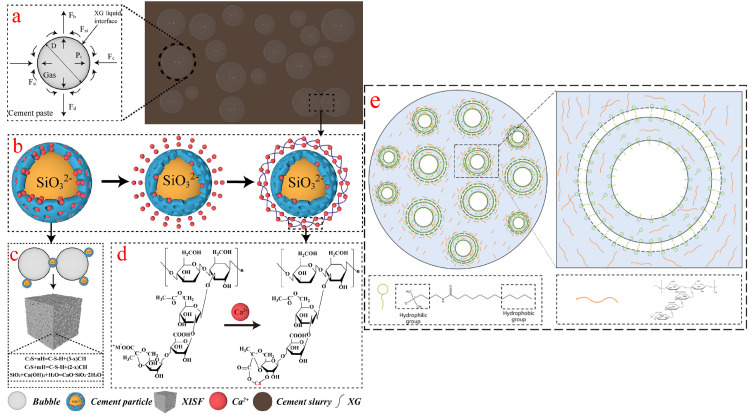
(**a**–**d**) Stabilization mechanism of XISF [170]. (**e**) Mechanism of CGA formation [171].

Through the coupling effect of organic surfactant and nanoparticles, She et al. modified the gas–liquid interface to prepare a new type of stable foam material. The preparation of the new foam material and the stability of the foamed concrete were studied experimentally. Results indicate that nanosilica and hydroxypropyl methyl cellulose enhance cell wall viscosity, slowing bubble coalescence and disproportionation by adsorption onto bubble surfaces. This prevents gas transfer between the gas and the liquid phase and physical drainage. The addition of organic surfactants and nanoparticles makes the pore structure more uniform and finer [169]. Zhao et al. [170] proposed an inorganic foam material with high stability, which was suitable for the top coal caving area and the closed partition wall of a coal mine. The gas–liquid interface was modified with xanthan gum (XG), and cement, fly ash, and other non-combustible inorganic materials were used as the basic material. A kind of highly stable inorganic curing foam (XISF) was synthesized and the effect of XG on foam stability was studied. Figure 11a–d show the stabilization mechanism of this ultra-stable prefabricated foam. The results show that when the XG content is 0.5 wt.%, the active functional groups in XG (such as hydroxyl and carboxylic) chelate with Ca2+ on the cement particles’ surface to form a “shell–core” structure, inhibit hydration, and improve the fire extinguishing performance of XISF.

Xu et al. [171] successfully prepared a colloid gas-foamed concrete (CGA) and cement slurry using a colloid gas foam material with a high water retention rate. Colloid gas-foamed concrete can form a hard oxygen barrier shell on the coal surface, thereby suppressing coal spontaneous combustion. Figure 11e shows the formation mechanism of CGA. The results indicate that colloid gas-foamed concrete demonstrates superior suppression of coal spontaneous combustion compared to traditional water-based foamed concrete, and the intersection temperature of treated coal is higher.

It can be seen from the above references that the current research on cement-based foam materials primarily focuses on (1) the development of foaming agents for water-based foam [149,150]; (2) the correlations among aggregates, mineral admixtures [153,172] and admixtures in substrates and the properties of materials such as compressive strength, pore structure, permeability, and thermal conductivity [173,174,175,176,177,178]; (3) application research of cement-based foaming materials in different engineering fields [179,180,181,182,183,184,185,186]. This is mainly because the main application field of cement-based foam materials remains in the building material and civil engineering industries, and studies on their characteristics and modification are carried out based on the application background. As for the utilization of cement-based foam material in coal mines to prevent coal spontaneous combustion, several key characteristics, including its solidified characteristics in the state of fresh foam fluid, the patterns of diffusion in complex fracture network, the cooling characteristics of concealed high-temperature fire sources, the characteristics of plugging air, the compressive resistance after solidification and the impact of pore structure on mechanical and thermal performance, need to be studied, and more systematic studies should be further conducted.

## 5. Conclusions

This paper systematically describes the application of foam materials in fire-fighting technologies. The formation mechanism, preparation schemes, chemical components and fire-fighting performance of those materials are emphasized. We also discussed the existing problems in the research on fire-fighting foam materials. This review will be of guiding significance for developing highly efficient coal fire prevention and control technologies, helping to ensure the safe mining of coal resources. The conclusions are as follows:

(1) Compared with two-phase foam, three-phase foam offers superior fire protection. Currently, research on the stability of three-phase foam and its seepage in porous media mainly focuses on the petroleum industry. Unlike the porous media of low permeability in oil reservoirs, fractured rock in mine goafs has less pressure and is mostly in a state of natural accumulation, and the seepage mode of three-phase foam is obviously different from that of foam fluid in primary reservoir rock. The existing studies on the three-phase foam used in coal mines mainly focus on experiments, and they mainly evaluate the fire-fighting performance of the foam. Further, other solid wastes, such as coal gasification slag, can also be considered for use as a component of the three-phase foam. The characteristics of the structural evolution of foam in porous media must be examined from a microscopic perspective for a full understanding. Further studies could provide new insights for predicting the seepage and diffusion behaviors of three-phase foam in porous media.

(2) Gel foam combines the benefits of gel and foam. At present, researchers are using advanced physical and chemical methods to develop a variety of effective gels and composite pastes with different inhibition mechanisms. More and more environmentally friendly and economical substrates and fillers have been used in the preparation of gel foams to reduce harm to the environment and human health. More attention has been paid to the mechanism of gelling, fire extinguishing efficiency, inhibition properties and mechanism of those new gel foams. However, the gel foam materials at present still have a few shortcomings, such as low foam expansion rate, long gel formation time and poor stability. Research on new gel foams is of great significance to further improve fire prevention and fire extinguishing performance.

(3) Sealing air leakage passages in coal mine goafs using curing foam to reduce oxygen contact with the coal seam is also a critical technology to prevent coal spontaneous combustion in goafs and high-risk areas. The studies on organic curing foams for mining mainly focus on the catalytic curing of resins and the toughening modification of foams. As a new type of goaf filling and plugging material, inorganic curing foam has the characteristics of isolating oxygen, cooling, and high compressive strength. However, it also has the problems of poor stability and weak accumulation ability. As for cement-based foam materials for preventing coal spontaneous combustion, there are several research directions, including the condensation characteristics in the state of fresh foam fluid, the pattern of diffusion in a complex fracture network, the heat insulation characteristics, the compressive resistance after solidification, and the influence of pore structure on mechanical and thermal properties.

## Figures and Tables

**Table 1:** Summary of preparation methods and properties of different types of gel foams.

Name	Components	Configuration Procedure	Thermal Stability	Inhibition Rate	Fire Extinguishing Performance	Ref.
New gel foam	(a) Compound foaming agent (sodium α-alkenyl sulfonate: fatty alcohol polyoxyethylene ether sodium sulfate = 8:4); (b) gelling agent; (c) organic crosslinking agent.	(1) Foaming solution + gelling agent (stirring) → Liquid A; (2) inorganic aluminum salt solution + ligand (stirring) → Liquid B; (3) Liquid A + Liquid B → Gel foam.	Not mentioned	The inhibition rate increased by 61.72% compared to two-phase foam.	No significant reignition of the coal body was observed.	[71]
Novel high-water-retaining foam	(a) Polymer composite (PC), a mixture of the microbial polysaccharide and galactomannan biopolymer; (b) organic boron complex; (c) foaming agent prepared from anionic surfactant and non-ionic surfactant.	(1) Foaming agent + water → base fluid (The concentration is 3 g/L); (2) polymer composite + base fluid (stirring about 15 min) → Dispersion liquid; (3) dispersion liquid + organic boron complex → uniform foaming solution; (4) mixed solutions + high-pressure air → high-water-retaining foam.	Not mentioned	Not mentioned	Within 30 min, the temperature of the burning coal dropped from approximately 700 °C to 34.7 °C.	[21]
Novel biomass sodium alginate gel foam	(a) Sodium alginate (SA); (b) calcium L-lactate (CL); (c) alkyl glycoside (APG); (d) tea saponin (TS).	(1) SA solution + 0.2 g APG + 0.1 g TS (Stir well) → Solution A; (2) dissolve CL + water → Solution B; (3) Solution A + Solution B (mechanical stirring) → biomass sodium alginate gel foam.	Not mentioned	The CO inhibition rate is 60.5% at 200 °C.	During the first 60 min, the coal sample temperature rapidly decreased from 965 °C to 90 °C, and after 200 min, it dropped to 30 °C.	[81]
PVA-H18 gel foam (PGF)	(a) Nanoparticles (hydrophobic); (b) sodium bicarbonate; (c) sodium tetraborate; (d) PVA.	(1) 3 g PVA + 100 mL deionized water (stirring) → PVA solution; (2) PVA solution + sodium bicarbonate (stirring) → PVA solution with PH 8.5; (3) 0.7 g nanosilica + foam + 0.4 g sodium tetraborate (stirring) → PGF.	Retain water for over 15 h at 100 °C.	The temperature of the coal sample dropped from 865 °C to 100 °C within 30 s.	Effectively preventing the recurrence of a coal fire after extinguishing.	[90]
XG/GG/HPAM gel foam	(a) Surfactants; (b) anionic polysaccharide xanthan gum (XG); (c) galactomannan guar gum (GG); (d) metal crosslinker; (e) gelling agent polyacrylamide (HPAM); (f) lab-made inhibitor.	Surfactant + XG + GG + HPAM + inhibitor (high-speed stirring) → XG/GG/HPAM gel foam.	The rate of water loss was 8.50% after heating at 100 °C for 1 h.	The inhibition rate at 100 °C is 74.48%.	Not mentioned	[82]
A novel foam gel	(a) Sodium metaborate tetrahydrate; (b) tetrabutyl titanate; (c) glucose monomer; (d) sodium bicarbonate (NaHCO_3_); (e) acrylic acid (AA); (f) acrylamide (AM); (g) potassium persulfate (KPS); (h) sodium hydroxide (NaOH); (i) commercially available N,N′-methylene bisacrylamide (MBA); (j) ethanol.	(1) Sodium acrylate + AM + crosslinking agent MBA + sodium bicarbonate (stirring)→Solution A; (2) sodium borate + initiator KPS + deionized water (stirring) → Solution B; (3) foaming agent + Solution B + prepared H-TiO_2_ (ultrasonication) → new solution B; (4) new Solution B + Solution A → Foam gel.	Not mentioned	The inhibition rate is 52.63%.	Has a marked inhibitory effect on smoke.	[84]
Temperature-resistant gel foam	(a) Acrylic acid (AA); (b) acrylamide (AM); (c) 2-Acryloylamino-2-methyl-1-propanesulfonic acid (AMPS); (d) N,N′-Methylenebisacrylamide (MBA); (e) ammonium persulfate (APS); (f) sodium hydroxide(NaOH); (g) calcium lignosulfonate (CLS).	(1) Acrylic acid + sodium hydroxide solution → Sodium acrylate solution; (2) AMPS + MBA+ CLS + sodium acrylate solution (stirring) → mixed solution; (3) APS+ mixed solution (stirring) → polymer solution.	Not mentioned	Not mentioned	Generated an enhanced mechanism with greater temperature resistance, stability and considerable potential application areas.	[91]
Highly stable double-crosslinked gel foam	(a) Fatty alcohol polyethylene ether sodium sulfate (AES); (b) polyether modified tri-siloxane (GT-248); (c) sodium alginate (SA); (d) carboxymethyl cellulose sodium (CMC); (e) ethylenediaminetetraacetic acid disodium (EDTA); (f) gluconate-δ-lactone (GDL).	(1) AES + GT-248 → foam agent (AG); (2) EDTA + CaCl_2_ + water (stirring) → EDTA-Ca solution; (3) 0.5 g SA + 0.05 g CMC + AG → SA/CMC thickener solution (left for 12 h) → SC solution; (4) SC solution+ EDTA-Ca+ GDL (thoroughly foamed) → double-crosslinked gel foam.	Not mentioned	The CO inhibition rate is 44.37% at 100 °C.	Extinguished the heat source within 470 s and reduced the temperature to 87 °C within 1300 s.	[92]
Environmentally friendly gel foam	(a) Alphaolefin sulfonate (AOS); (b) alkyl ethoxy polyglycosides (AEG); (c) sodium silicate; (d) sodium bicarbonate.	(1) Foaming agents: AOS and AEG; (2) gelling agents: sodium silicate; (3) crosslinking agents: sodium bicarbonate.	Thermal stability depends on the formulation.	Not mentioned	As the concentration of NaHCO_3_ increases, the fire extinguishing performance improves.	[22]
An environmentally friendly antioxidant foamed gel	(a) Modified sodium polyacrylate (MSP); (b) konjac glucomannan; (c) sodium dodecyl sulfate; (d) sodium alpha-olefin sulfonate; (e) modified silicone polyether microemulsion; (f) montmorillonite; (g) tert-butyl hydroquinone (TBHQ).	(1) Crosslinking agent + foaming agent + foam stabilizer + deionized water → mixed solution; (2) MSP + montmorillonite/TBHQ → slowly mixed; (3) stirred for 5 min.	Suppressed the thermal decomposition stage after 300 °C.	Inhibit the oxidation reaction of coal.	Prevents coal from contacting oxygen. Antioxidant components mitigate chemisorption and chemical reactions.	[93]
SA-Ca^2+^@TA-GF	(a) Tannic acid (TA) and calcium L-lactate (CL); (b) sodium alginate (SA); (c) composite foaming agent (CFA).	(1) TA + CFA + SA solution → mixed solution; (2) CL+ mixed solution (mechanical stirring) → gel foam.	Not mentioned	At 200 °C, the inhibition rate is 79.6%.	Within 20 min, the temperature of the coal decreased rapidly from 965 °C to 98.8 °C.	[85]
Biomass gel foam	(a) Carboxymethyl chitosan (CMCS); (b) composite crosslinking agent (CCA); (c) composite foaming agent (CFA) was a combination of anionic and non-ionic surfactants with a 1:1 ratio; (d) foam stabilizer agent (FSA).	(1) CMCS + water → CMCS solution; (2) CMCS solution + CFA + FSA + CCA (high-speed stirring) → biomass gel foam.	The water-holding rate was 49.34% after heating for 10 h at 80 °C.	The CO inhibition rate is 67.43% at 100 °C.	Excellent flame-retardant properties.	[94]
New eco-friendly gel foam based on biomass pectin material (LMP-Ca)	(a) Low methoxyl pectin (LMP); (b) calcium L-lactate (Ca-L); (c) Biomass compound foaming agent (BF) was composed of tea saponin (TS) and alkyl glycoside (APG).	(1) BF solution + LMP solution (mechanically stirred) → foaming solution; (2) Ca-L solution + foaming solution (fully stirred) → LMP-Ca.	Not mentioned	The CO inhibition rate is 72.1% at 180 °C.	The temperature of the coal decreased from 960 °C to 60 °C within 20 min, with no reignition occurring.	[87]
Gel-stabilized foam	(a) Thickening agent (TA); (b) crosslinking agent (CA); (c) foaming agent (FA) composed of the surfactant compound.	(1) TA + CA + FA + water → uniform foaming solution; (2) gel-stabilized foam.	Not mentioned	The foam can form a dense covering film with an excellent oxygen barrier	Not mentioned	[95]
PE/SA-Ca	(a) Pectin (PE); (b) sodium alginate (SA); (c) calcium L-lactate (Ca-L); (d) biomass foaming systems (BS).	(1) 0.2 wt.% APG + 0.1 wt.% TS (stirring) → biomass foaming system BS; (2) Ca-L + water → Ca-L crosslinking agent solution; (3) PE solution + SA solution → mixed solution of SA/PE; (4) BS + SA/PE solution + Ca-L crosslinking agent solution (stirring fully) → PE/SA-Ca.	Maintains membrane integrity under high temperature.	The CO inhibition rate is 78.06% at 180 °C.	Within 20 min, the coal fire temperature was reduced from 960 °C to 68.9 °C, effectively preventing coal dust reignition.	[96]
CPSF	(a) Fly ash (FA); (b) sodium alginate (SA); (c) sodium dodecyl sulfate (SDS).	(1) FA particles+ SA solution→stable gel suspensions (SGS); (2) 15 parts of SDS+ 100 parts of water (stirring) → foaming solution; (3) SGS + foaming solution (stirred fully) → CPSF.	Not mentioned	Not mentioned	It adheres well to the coal particle surface, significantly delays the onset time of CO, and shows good inhibition performance.	[97]
Silica gel foam	(a) Foaming agent: sodium dodecyl sulfate (SDS); (b) gel agent: sodium polyacrylate; (c) crosslinking agent: konjac gum; (d) foam stabilizer: xanthan gum; (e) nanosilica; (f) antioxidant: tert-butyl hydroquinone (TBHQ); (g) modification reagent: montmorillonite.	(1) Foaming agent + foam stabilizer + crosslinking agent + gel agent → gel foam state; (2) acrylic acid + potassium persulfate → modified montmorillonite (O-MMT); (3) modified montmorillonite (cause free radical reaction) → antioxidant system; (4) gel foam state + antioxidant system (fully mixed) + nanoscale particles → silica gel foam.	Not mentioned	Within the range of 60 to 100 °C, the concentration of free radicals shows a marked downward trend.	Modified nanosilica particles and antioxidants can enhance the suppression efficiency of foam liquid films while improving their mechanical strength and stability.	[98]

**Table 2:** Comparison of properties of organic curing foams.

Species	Raw Materials and Forms	Advantage	Disadvantage	Refs.
Polyurethane foam	Polyether polyols, isocyanates, diffusion crosslinkers, foaming agents, catalysts and flame retardants are expanded and cured according to a certain proportion.	Good viscoelasticity and stability, good sealing effect.	Polyurethane is a flammable substance, producing a lot of toxic smoke in case of fire. At the same time, the foaming reaction releases more heat, resulting in higher production costs.	[110,111,112].
Phenolic foam	Using phenolic resin as a substrate, then adding curing agent and foaming agent, closed-cell foam material is formed after a chemical reaction, which is mainly used in filling and sealing wall construction in high-volume areas of coal mines.	It overcomes the disadvantages of large heat release and inflammability, low thermal conductivity, while having a short reaction time, good adiabatic performance and high expansion rate, and it can be used continuously at 140 °C–160 °C.	It is prone to breaking, and phenolic substances are toxic and can form carcinogens. The foam is brittle and powdery. Compared with polyurethane foam, the bonding force is weaker and the cost is higher.	[113,114,115,116].
Urea–formaldehyde foam	It is a polymer foam material formed by chemical or physical foaming under the action of a foaming agent and hardener, with urea–formaldehyde resin as the base material.	Lightweight, high expansion ratio, no heat transfer, non-combustible and low reaction heat release temperature, low production cost, only half of the cost of phenolic foam.	Low strength, weak bearing capacity. Irritant gas is released during the reaction and pollutes the working environment.	[117,118,119].
Cured polymer composite foam	Synthetic with various types of raw materials.	Depending on the material used and the preparation method, different composite foams have different characteristics.	The mechanical properties are different from those of conventional filling materials, and each material needs to be studied and analyzed.	[120,121].

## Data Availability

No new data were created or analyzed in this study. Data sharing is not applicable to this article.
